# Acute Suppurative Thyroiditis (AST) With Thyroid Abscess: A Rare and Potentially Fatal Neck Infection

**DOI:** 10.7759/cureus.29062

**Published:** 2022-09-11

**Authors:** Vivek Sanker, Azeem Mohamed, Chaithra Jadhav

**Affiliations:** 1 General Surgery, Noorul Islam Institute of Medical Science (NIMS) Medicity, Trivandrum, IND; 2 Pathology, Indira Gandhi Medical College and Research Institute (IGMCRI), Puducherry, IND

**Keywords:** pyriform sinus fistula, incision and drainage of abscess, hypothyroidism, acute suppurative thyroiditis, thyroid abscess

## Abstract

Thyroid abscess, although rare, is a condition that usually occurs as a sequela of acute suppurative thyroiditis (AST) which is an infection of the thyroid gland. The infrequent occurrence of thyroid abscess is due to the unique anatomical and physiological characteristics of the gland which renders it resistant to infections. Delay in diagnosis and treatment can have adverse outcomes and serious complications such as septicaemia, descending necrotising mediastinitis, extension into deep spaces of the neck, and tracheal or oesophageal perforation. The mainstay of management is a combination of systemic antibiotics along with incision and drainage, and rarely surgery. We report the case of a 37-year-old male with acute suppurative thyroiditis with a thyroid abscess. He underwent incision and drainage of the abscess and was subsequently treated with systemic antibiotics. This condition warrants a prompt and timely diagnosis with appropriate management as it often leads to fatal complications if not diagnosed early.

## Introduction

Acute suppurative thyroiditis (AST) leading to thyroid abscess is an uncommon clinical entity owing to its unique features which confer its protection from infections [1]. It includes rich blood supply and lymphatic drainage, high iodine content, and a thick capsule that encloses the gland and hence separates it from the surrounding structures [2]. Thyroid abscess and AST represent only 0.1 to 0.7% of surgically treated thyroid pathologies [3]. The most common etiological agent associated with AST is bacteria and usually polymicrobial. Fungal agents such as *Coccidioides immitis*, Aspergillus, and Actinomyces can cause infection in immunocompromised patients. Infection of the gland is usually following bacterial spread through blood and the left lobe is more frequently involved [4].

## Case presentation

A 37-year-old male presented to the emergency department with complaints of fever and painful neck swelling of two days duration. His past history is significant for pyogenic meningitis, septicaemia, liver abscess with multiple pulmonary nodules, two months back, and also for type 2 diabetes mellitus. No history of allergies or any previous surgeries was recorded. The patient was initially admitted to the department of otorhinolaryngology for evaluation. On clinical examination of the patient, tenderness present on the midline below the thyroid prominence and right upper jugular lymphadenopathy was also noted. Diffuse swelling was present over this area. Initial diagnostic assessment of the patient showed an elevated total white blood cell (WBC) count, increased erythrocyte sedimentation rate (ESR), and increased acute phase reactants such as C-reactive protein (CRP) and procalcitonin (Table 1). 

**Table 1:** Initial laboratory test results

Blood Investigations	Patient Results	Reference Value
Fasting Blood Sugar	210 mg%	70 – 99 mg%
Glycated Haemoglobin (HbA1C)	9.6%	<5.7%
Complete Blood Count (CBC)		
Haemoglobin (Hb)	7.2 gm%	11 – 17 gm%
Total White Blood Cell (WBC) count	20210 cells/cumm	4000 – 11000 cells/cumm
Platelet Count	2.22 lakhs/cumm	1.5 – 5 lakhs/cumm
Erythrocyte Sedimentation Rate (ESR)	110 mm/hr	0 – 20 mm/hr
Renal Function Test		
Urea	25 mg%	20 – 40 mg%
Creatinine	0.5 mg%	0.6 – 1.3 mg%
Liver Function Test		
Total Bilirubin	0.94 mg%	0.20 – 1.30 mg%
Indirect Bilirubin	0.9 mg%	0.20 – 1.10 mg%
Alanine transaminase (ALT)	19 IU/L	< 50 IU/L
Aspartate aminotransferase (AST)	17 IU/L	15 – 37 IU/L
Alkaline phosphatase (ALP)	83 IU/L	50 – 136 IU/L
Serum Electrolytes		
Sodium	133 mEq/L	136 – 145 mEq/L
Potassium	4.2 mEq/L	3.5 – 5.1 mEq/l
Thyroid Function Test		
Thyroid Stimulating Hormone (TSH)	0.14 uIU/mL	0.25 – 5.60 uIU/mL
Total T3	1.61 ng/mL	0.50 – 1.78 ng/mL
Total T4	8.41 ug/dL	4.50 – 12.23 ug/dL
Acute Phase Reactants		
C-reactive protein (CRP)	179.6 mg/L	<5mg/L
Procalcitonin	5.87 ng/mL	>2 ng/mL: High risk of severe sepsis

**Figure 1:**
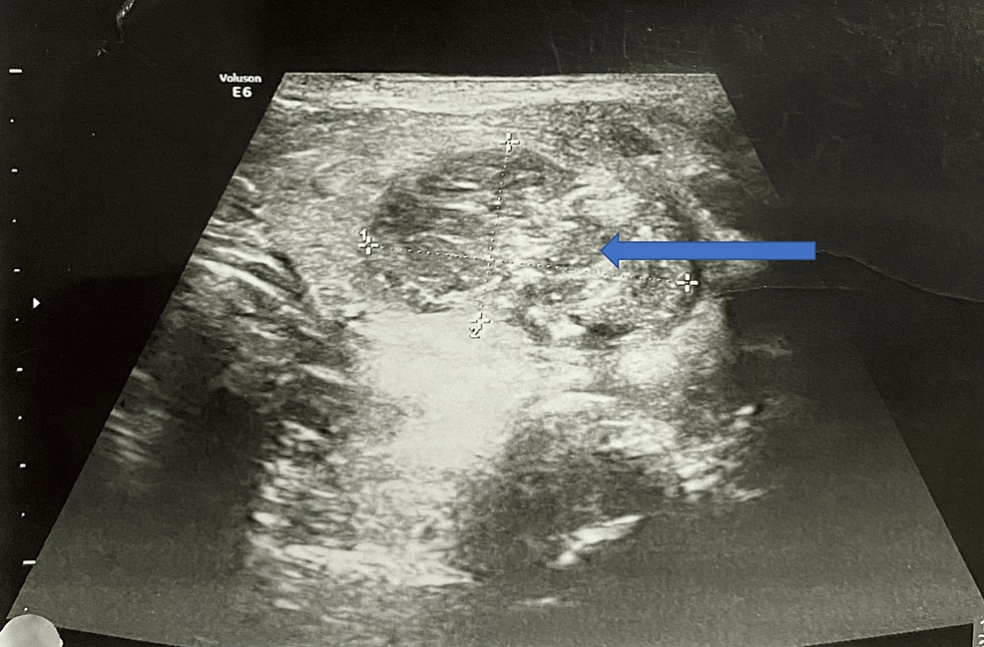
Ultrasonography image of neck/thyroid Ultrasonography of neck/thyroid showing heterogeneous nodule measuring 28 × 18 mm in the left lobe with internal calcification (shown in blue arrow). The left thyroid lobe appears to be heterogeneous with increased vascularity.

Ultrasonography (USG) of the neck /thyroid showed the left thyroid lobe to be heterogeneous with increased vascularity. Heterogeneous nodule measuring 28 × 18 mm in the left lobe with internal calcification. Increased peripheral vascularity was noted. Adjacent sternocleidomastoid muscle appears edematous and surrounding fat appeared inflamed. These features are suggestive of left acute suppurative thyroiditis with nodules (Figure 1). Differential diagnosis: nodular goiter with secondary infection. USG of abdomen and pelvis showed enlarged liver measuring 16.7 cm in size with fatty changes in parenchyma suggestive of hepatomegaly with grade I fatty changes.

**Figure 2:**
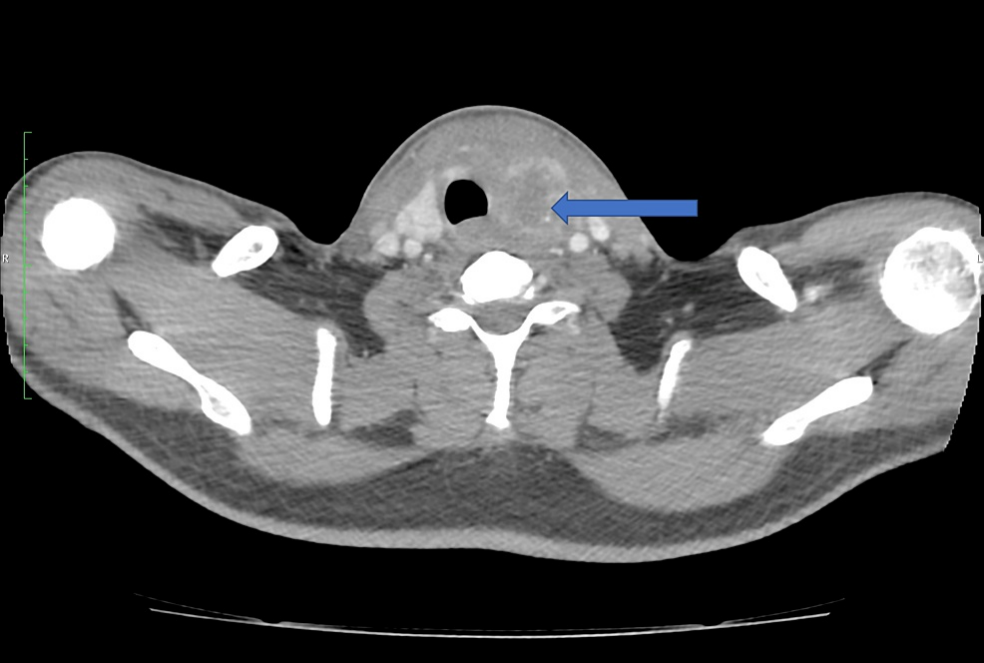
CECT image of the neck Contrast-enhanced computed tomography (CECT) neck showing thyroiditis with nodule and a large ill-defined hypo-dense lesion in the left lobe of the thyroid, extending to the isthmus, suggestive of an evolving abscess within the thyroid nodule (shown in blue arrow).

Subsequently, contrast-enhanced computed tomography (CECT) scan of the neck and high resolution computed tomography (HRCT) chest were done. CECT neck showed a large ill-defined hypo-dense lesion in the left lobe of the thyroid, extending up to the isthmus approximately measuring 4.5 x 2.8 x 3.5 cm suggestive of an evolving abscess within the thyroid nodule (Figure 2). It is seen to reach a depth of 2.5 cm from the skin surface and is pushing the trachea and esophagus towards the right.

**Figure 3:**
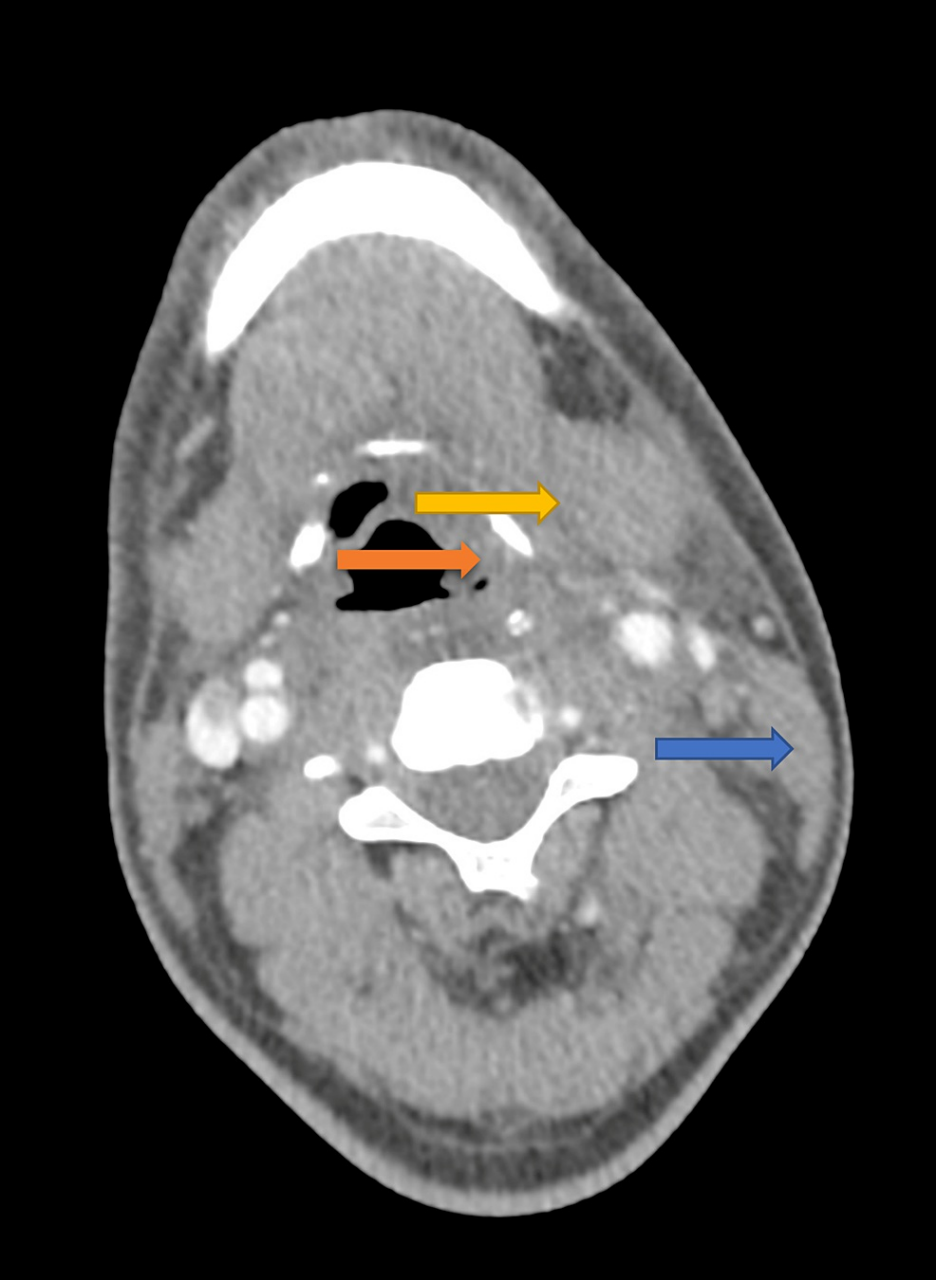
CECT image of neck/thorax Contrast-enhanced computed tomography (CECT) neck/thorax showing left aryepiglottic fold asymmetrically bulky (shown in orange arrow), left sternocleidomastoid muscle appears bulky and edematous (shown in blue arrow), and obliteration of the left vallecula (shown in yellow arrow).

A hypo-dense area was noted extending from the posterior aspect of the collection extending towards the left lateral wall of the upper oesophagus which was suspicious of a tiny fistulous tract. A nodule with peripheral calcifications was noted in the left lobe of the thyroid measuring 2 x 1.6 cm seen adjacent to it. The subcutaneous fat stranding was noted in the anterior neck extending to the anterior upper chest well. The left aryepiglottic fold was asymmetrically bulky and the adjacent sternocleidomastoid muscle also appeared bulky and edematous (Figure 3). 

**Figure 4:**
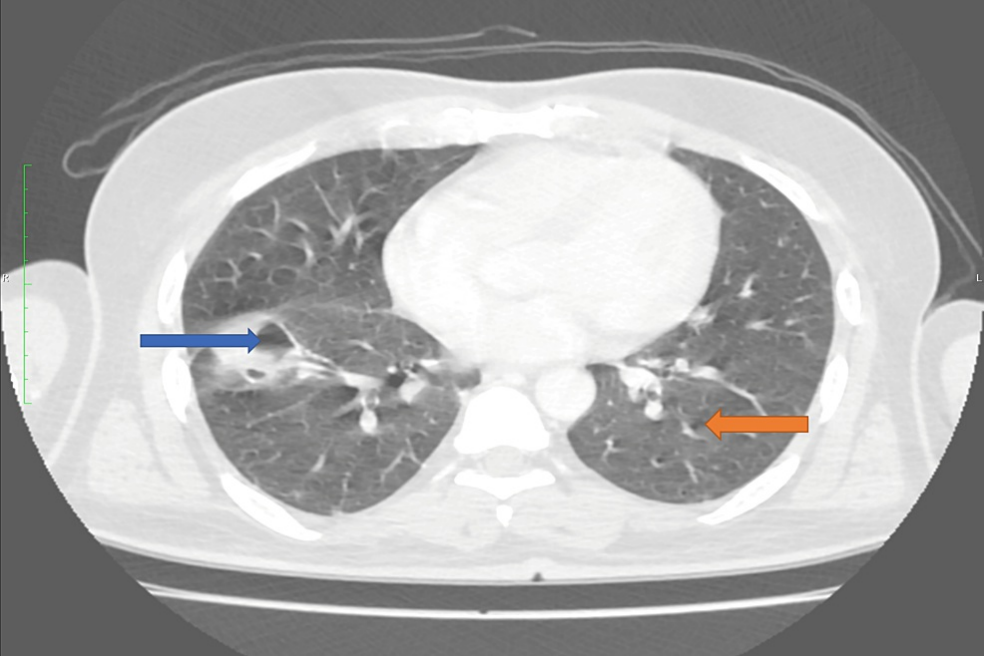
HRCT chest image High-resolution computed tomography (HRCT) chest showing cavitating nodules in bilateral lung fields, the largest measuring 3.9 x 2.5 cm in the antero-basal segment of the right lobe (shown in blue arrow).  Multiple small solid nodules were noted in bilateral lung fields, the largest measuring 7 mm in the superior segment of the left lower lobe (shown in orange arrow).

HRCT chest showed cavitating nodules in bilateral lung fields, the largest measuring 3.9 x 2.5 cm in the antero-basal segment of the right lobe, abutting oblique fissure, suggestive of septic emboli. Multiple small solid nodules were noted in bilateral lung fields, the largest measuring 7 mm in the superior segment of the left lower lobe. The subcutaneous fat stranding was noted in the anterior neck extending to the anterior upper chest wall and also in the superior mediastinum. Multiple sub-centimetric mediastinal lymph nodes were noted, the largest measuring 7 mm in the right lower paratracheal region (Figure 4).

Subsequently, general surgery consultation was taken and the patient was transferred to the surgical side for further management. He underwent incision and drainage of thyroid abscess under general anaesthesia. Kocher's collar incision was made, sub-platysmal flap raised, deep fascia opened in the midline, findings were confirmed, abscess cavity incision was done and pus drained. The pus sample was sent for culture and sensitivity and the nodule was sent for biopsy.

During the initial post-operative period, the patient was shifted to the surgical intensive care unit and was on mechanical ventilation as he showed signs of respiratory distress and was not maintaining saturation. Subsequently, the patient improved clinically and was extubated and shifted to the post-operative intensive care unit. Later pulmonology and endocrinology consultations were taken. Pus culture revealed the growth of *Klebsiella pneumoniae. *Infectious disease (ID) consultation was taken and the patient was started on ciprofloxacin and ceftriaxone-tazobactam combination for two weeks. The antibiotic sensitivity report of the patient is as follows (Table 2).

**Table 2:** Antibiotic sensitivity test results

Antimicrobial	Minimum Inhibitory Concentration (MIC)	Interpretation
Amoxicillin/Clavulanic Acid	< 2	Sensitive
Piperacillin/Tazobactam	< 4	Sensitive
Cefuroxime	2	Sensitive
Ceftriaxone	< 0.25	Sensitive
Cefoperazone/Sulbactam	< 8	Sensitive
Cefipime	< 0.12	Sensitive
Amikacin	< 1	Sensitive
Gentamicin	< 1	Sensitive
Ciprofloxacin	< 0.06	Sensitive
Trimethoprim/Sulfamethoxazole	< 20	Sensitive

**Figure 5:**
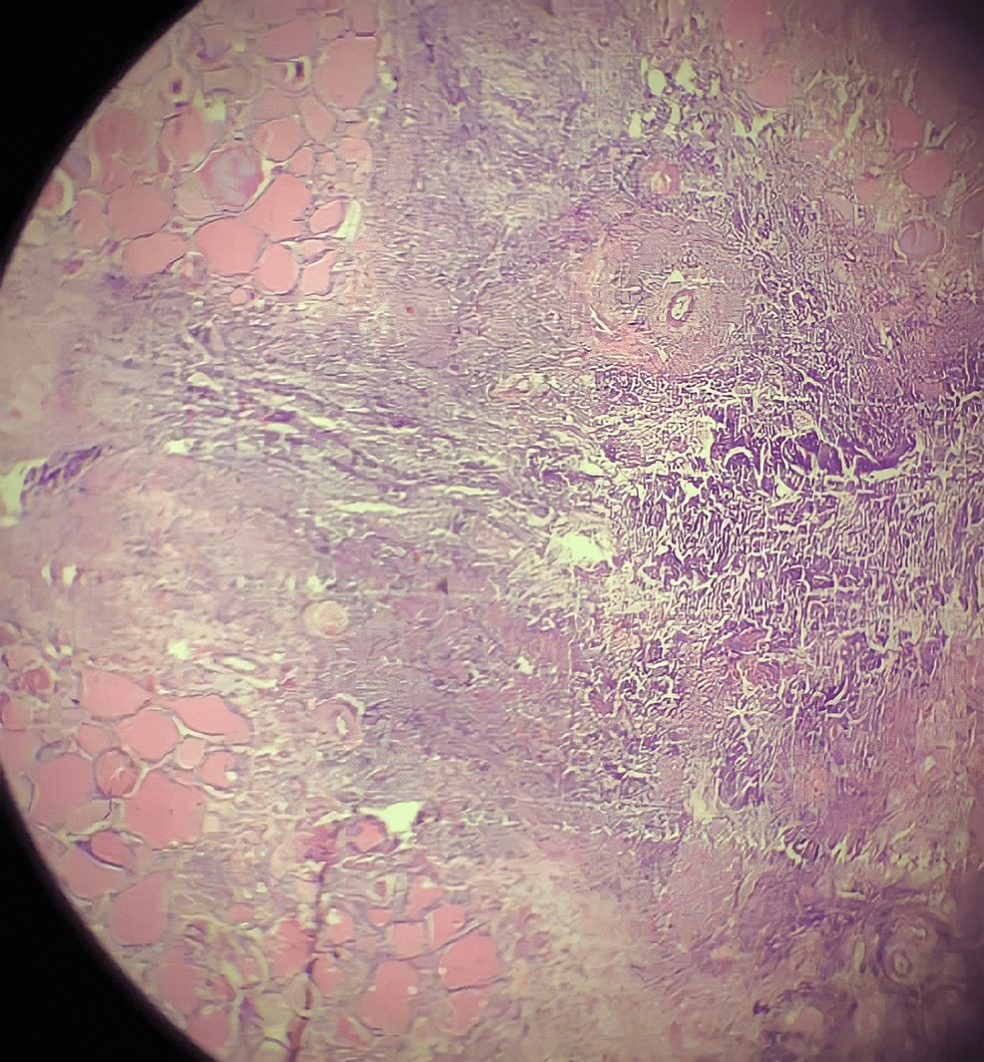
Histopathological image The section shows thyroid tissue with dense neutrophilic infiltration forming an abscess (H&E, 40x)

Daily dressing of the wound was done and the patient was discharged as he got symptomatically better. Biopsy of the operative specimen from within the thyroid nodule showed a dense infiltrate of inflammatory cells forming micro abscess and is composed of sheets of neutrophils along with necrotic debris which is also surrounded by fibrosis and congested blood vessels. The inflammatory infiltrates are also seen surrounding, destroying, and replacing thyroid follicles. The findings are suggestive of acute suppurative thyroiditis with abscess formation (Figure 5). The biopsy sample did not show any evidence of a calcific nodule, so a repeat CT scan and follow-up are planned for a subsequent visit to reevaluate the same. 

## Discussion

Acute Suppurative Thyroiditis (AST) is a rare and fatal emergency condition of the thyroid gland which can lead to abscess formation as a sequelae [5]. It can further result in deadly complications such as necrotising mediastinitis, extension into deeper neck spaces, and perforation of the trachea or esophagus [6]. The majority of the patients affected are under the age of 40 years, with a slight female predominance [7].

The age of the index patient is the same as the usual age of presentation of this condition. Among adult patients with acute suppurative thyroiditis, there is usually evidence of pre-existing thyroid diseases such as Hashimoto’s thyroiditis or thyroid malignancy. Children with congenital anatomical anomalies such as pyriform sinus fistula and 3rd or 4th branchial pouch canal [8,9] are at increased risk of acquiring AST. Most cases involve the left thyroid lobe, lymphatic and hematogenous routes being the common modes of spread of the infection [4].

AST and thyroid gland abscess are clinically uncommon conditions owing to the anatomical and physiological characteristics of the gland which attributes to its resistance to infections. These include rich blood supply and lymphatic drainage, high iodine content, and a thick capsule encasing the gland from its surrounding [2,10,11]. Common clinical manifestations include fever, neck swelling, sore throat, difficulty in swallowing, and pain similar to how the index case presented with. Localised lymphadenopathy is also commonly observed. Rarely, it can present as an endocrine emergency, with features of systemic thyrotoxicosis, secondary to the release of thyroid hormones following gland destruction by bacterial invasion. Most cases have a bacterial etiology, among which *Staphylococcus aureus* is the predominant cause. Others include Streptococcus and Salmonella. Mycobacterium can rarely be involved, and fungal agents have been found to be the causative agent among immunocompromised patients [12].

Early and prompt diagnosis is crucial in deciding the prognosis of patients with AST and thyroid abscess. Often the diagnosis is delayed as the clinical presentations are subtle with or without serious outcomes. It can also mimic non-infectious inflammatory thyroid conditions and need to be differentiated from conditions such as De Quervain’s thyroiditis, goiter, adenoma, and thyroid malignancy.

Clinical examination, radiological investigations along with histopathological findings help to make the diagnosis. Routine blood investigations with the exception of leukocytosis and elevated acute phase reactants are usually normal [13]. Most patients are also euthyroid similar to the presentation of the index case [14]. The initial radiological investigations performed are X-ray and USG. The common USG findings of AST include hypoechoic lesions within the gland, accumulation of surrounding fluid, and heterogeneous areas [15]. In the index case, findings include a heterogeneous left thyroid lobe with increased vascularity, a heterogeneous nodule measuring 28 × 18 mm in the left lobe with internal calcification, and increased peripheral vascularity.

Computed Tomography (CT) scan and Magnetic Resonance Imaging (MRI) are also done in many cases. In this case, a CECT scan of the neck and upper chest revealed a large ill-defined hypo-dense lesion in the left lobe of the thyroid suggestive of an evolving abscess within the left thyroid gland. A hypo-dense area was also noted extending from the posterior aspect of the collection extending towards the left lateral wall of the upper oesophagus which is suspicious of the tiny fistulous tract.

A biopsy is always conclusive and in our case also the findings were suggestive of acute suppurative thyroiditis with abscess formation. Treatment involves systemic antibiotic therapy along with incision and drainage. The pus aspirated should be sent for culture and sensitivity to determine the appropriate antibiotics and the tissue sample for histopathological examination to confirm the diagnosis and rule out malignancy [16]. Persistence of collection, worsening of symptoms, recurrence, and extensive necrosis may require surgical lobectomy of the involved thyroid gland [17]. In patients with severe thyroiditis, gland destruction can result in hypothyroidism and require timely follow-up with a thyroid function test [18].

## Conclusions

Thyroid abscess is an uncommon clinical entity to come across in day-to-day clinical practice. As it is commonly associated with underlying thyroid diseases in adults and congenital anatomical defects in children, such conditions should also be ruled out. Radiological investigations help to assess the extent of involvement and the site, which is crucial for the drainage of the abscess. This entity often leads to complications and deadly outcomes if not diagnosed early and treated adequately. Thus it should be thought of as a differential diagnosis in cases of neck swelling.
